# Specific Relationship between Excitatory Inputs and Climbing Fiber Receptive Fields in Deep Cerebellar Nuclear Neurons

**DOI:** 10.1371/journal.pone.0084616

**Published:** 2014-01-08

**Authors:** Fredrik Bengtsson, Henrik Jörntell

**Affiliations:** Neural Basis for Sensorimotor Control, Department of Experimental Medical Science, Lund University, Lund, Sweden; The Research Center of Neurobiology-Neurophysiology of Marseille, France

## Abstract

Many mossy fiber pathways to the neurons of the deep cerebellar nucleus (DCN) originate from the spinal motor circuitry. For cutaneously activated spinal neurons, the receptive field is a tag indicating the specific motor function the spinal neuron has. Similarly, the climbing fiber receptive field of the DCN neuron reflects the specific motor output function of the DCN neuron. To explore the relationship between the motor information the DCN neuron receives and the output it issues, we made patch clamp recordings of DCN cell responses to tactile skin stimulation in the forelimb region of the anterior interposed nucleus *in vivo*. The excitatory responses were organized according to a general principle, in which the DCN cell responses became stronger the closer the skin site was located to its climbing fiber receptive field. The findings represent a novel functional principle of cerebellar connectivity, with crucial importance for our understanding of the function of the cerebellum in movement coordination.

## Introduction

Recent studies of deep cerebellar nuclear (DCN) neurons have focused exclusively on the integration of inhibitory Purkinje cell input and the postinhibitory rebound excitation [1]–[4]. But the primary response mode of DNC neurons under behavior is excitatory modulation that arise without any substantial preceding inhibition [5]–[8], and which are therefore likely to be a least partly driven by the mossy fiber (MF) inputs to DCN neurons. However, there is currently very limited understanding of the potential contributions of the excitatory MF inputs to DCN cells *in vivo* and the functional relationship between the MF input and the DCN output has not been explored at all.

The DCN cells of the forelimb region of the anterior interposed nucleus (AIP) are innervated by the Purkinje cells (PCs) of the C1 and C3 zones [9], [10]. The PCs of the C1 and C3 zones are organized in microzones in which the PCs have specific cutaneous receptive fields for climbing fiber (CF) and parallel fiber (PF) inputs [11]–[14]. The PCs of a microzone converge on the same local group of cells in the anterior interposed nucleus, and thus form a cerebellar cortico-nuclear microcomplex [1], [10], [15], [16]. Importantly, each microcomplex control a specific synergy [17] and target specific regions of the motor cortex [18]. Microcomplexes therefore seem to correspond to the functional units of the cerebellar circuitry. Consequently, the CF receptive field of a DCN neuron is a tag to its specific motor function.

The DCN neurons receive MF input from a set of spinocerebellar and spino-reticulocerebellar tracts (SCTs and SRCTs, respectively) [19], which originate from spinal neurons [20], [21]. Some components of these pathways are strongly activated by cutaneous input [22] and have specific receptive fields [23], similar to CFs [24]. The information carried by the SCT/SRCT pathways are derived from spinal interneurons and represent activity from the spinal motor control circuitry [19]. Importantly, the specific receptive field of a spinal neuron reflects its specific function in terms of muscle control [25], [26]. Consequently, the location of the receptive field of a spinal neuron is a tag to trace its specific motor function.

Hence, for the DCN neuron, analysis of the receptive fields of the MF input and its relationship to the location of the CF receptive field essentially describe the relationship between the motor information the DCN neuron receives and the motor information it issues. During ongoing motor control, this relationship translates to that the activation of specific spinal synergy controllers (i.e. spinal premotor interneurons [27]) will lead to simultaneous activation of certain DCN neurons, which in turn will result in an increased drive on specific sets of synergies determined by the sets of upper motor neurons (i.e. in the motor cortex and the red nucleus) that the DCN cell innervates. The relationship could be important for determining how cerebellar movement coordination works. To explore this relationship, we made whole cell intracellular recordings and loose cell-attached extracellular recordings from DCN cells of the AIP *in vivo* and recorded their responses to localized tactile stimulation of various skin sites. Our findings suggest that the MF input and the output of the DCN cell are functionally matched.

## Materials and Methods

### Ethics Statement

The experimental procedures were approved in advance by the Malmö/Lund Animal Research Ethics Committee (permit number and approval-ID: M32-09). Initial surgery was performed under propofol anesthesia, and all efforts were made to minimize suffering. Our EEG recordings were characterized by a background of periodic 1–4 Hz oscillatory activity, periodically interrupted by large-amplitude 7–14 Hz spindle oscillations lasting for 0.5 s or more. These forms of EEG activities are normally associated with deep stages of sleep [28]. The pattern of EEG activity and the blood pressure remained stable and did not change with noxious stimulation throughout experiments.

Adult cats were prepared as previously described [12], [29]. Briefly, following an initial anesthesia with propofol (Diprivan® Zeneca Ltd, Macclesfield Cheshire, UK), the animals were decerebrated at the intercollicular level and the anesthesia was discontinued. The animals were artificially ventilated and the end-expiratory CO_2_, blood pressure and rectal temperature were continuously monitored and maintained within physiological limits. Mounting in a stereotaxic frame, drainage of cerebrospinal fluid, pneumothorax and clamping the spinal processes of a few cervical and lumbar vertebral bodies served to increase the mechanical stability of the preparation.

### Recordings and Stimulation

The initial delineation of the forelimb area of the C3 zone in the cerebellar anterior lobe and the continuous monitoring of the general condition in the sensitive mossy fiber-to-granule cell-to-parallel fiber pathway were performed as described previously [13]. Also the procedure for placing recording and stimulation electrodes in the lateral reticular nucleus (LRN) have been described in detail elsewhere [1], [23]. In vivo patch clamp recordings were made from deep cerebellar nuclear cells with patch pipettes pulled to 6–14 MOhm (potassium-gluconate based internal solution, chloride 7.3 mM, same solution as in Bengtsson et al. [1],Jorntell and Ekerot [29], [30]). A special adaptation for the DCN recordings was to pull the final 10 mm of the patch pipettes to one long, narrow part (<200 um) using a custom-made box-filament on a Sutter micropipette puller (P-97, Sutter Instruments Co., USA). Extracellular metal electrode recordings (exposed metal tips 3–15 um) were made from DCN neurons, Purkinje cells of the C3 zone, and CF field potentials in the molecular layer of the C3 zone.

In order to localize the anterior interposed nucleus (AIP), all experiments started with a topographical exploration using a metal electrode. The microelectrode was inserted at around the border between the C2 and C3 zones just rostral to the primary fissure at 90° angle relative to the horizontal stereotaxic plane. To keep track of the electrode location, we continuously monitored the spontaneous activity and the field and unitary responses evoked by electrical skin stimulation throughout electrode tracks. The dorsal border of the AIP was identified by a marked increase in background noise (presumably reflecting neuronal multi-unit activity) relative to the overlying white matter, and the characteristic field potentials evoked by electrical skin stimulation [10], [17]. The medial and lateral borders of the AIP could be identified on the basis of the receptive field topography for CF-activated Purkinje cell inputs, as previously described [10], [17], [18]. The ventral border of the nucleus was characterized by a reduction in background noise.

Patch clamp pipettes were lowered under high positive pressure (3–10 atmospheres) until we reached the dorsal part of the nucleus according to the previous identification done with the metal electrode. Once inside the nucleus, the positive pressure was reduced but not removed. When dramatic increases in tip resistance occurred as the electrode was advanced, the positive pressure was removed and a seal formation was attempted. Once obtained (0.5–6 GOhm), rapid application of negative pressure was used to gain access to intracellular space. Major quality checks for the whole cell recordings were the spike amplitudes (at least 35 mV) and the amplitudes of the spontaneous and IO-evoked giant IPSPs. Resting membrane potential was defined as the average potential recorded between spikes at 0 pA bias current within 30 s after intracellular access (−50 to −55 mV). Whenever spike amplitudes or giant IPSP amplitudes (see Bengtsson et al. [1]) deteriorated by more than 20% from the initial record after we gained intracellular access, the recording was stopped since it was taken as an indication that the seal between the recording electrode and the cell membrane deteriorated.

Input from the skin was evoked using a strain-gauge device mounted on the index finger of the investigator and pairs of closely spaced percutaneous needle electrodes (stimulated with one square pulse at 0.1 ms, 1 Hz, 1 mA) [29], [30]. Quantification of evoked responses was made from averaged intracellular responses and from peristimulus histograms of spike responses. Data in peristimulus histograms were obtained after subtracting the prestimulus baseline activity (an average of the activity 200 ms before the stimulation) from the response. In some cases the prestimulus baseline activity was subtracted from the histogram to obtain ‘net response’ histograms. Responses evoked by manual skin stimulation were defined as starting at the first bin after the onset of the stimulation that exceeded the baseline by at least 1.5 standard deviations and lasting for 50 ms after the onset. Data were obtained by repeated stimulation (at 0.5–1 Hz) to the same skin site 30–60 times. For each cell recorded, we quantified the input from 8–18 different skin sites.

All data are given as mean ±1 standard deviation unless stated otherwise.

## Results

We made intracellular whole cell recordings (N = 8) as well as cell-attached extracellular recordings (N = 104) from cells in the forelimb area of the AIP, an area that we have previously characterized with respect to its general topography [10], [17], [18]. All cells in the present study were classified as putative excitatory projection neurons based on the same analysis of interspike intervals as in our recent paper focusing on inhibitory DCN cell responses [1].

**Figure 1:**
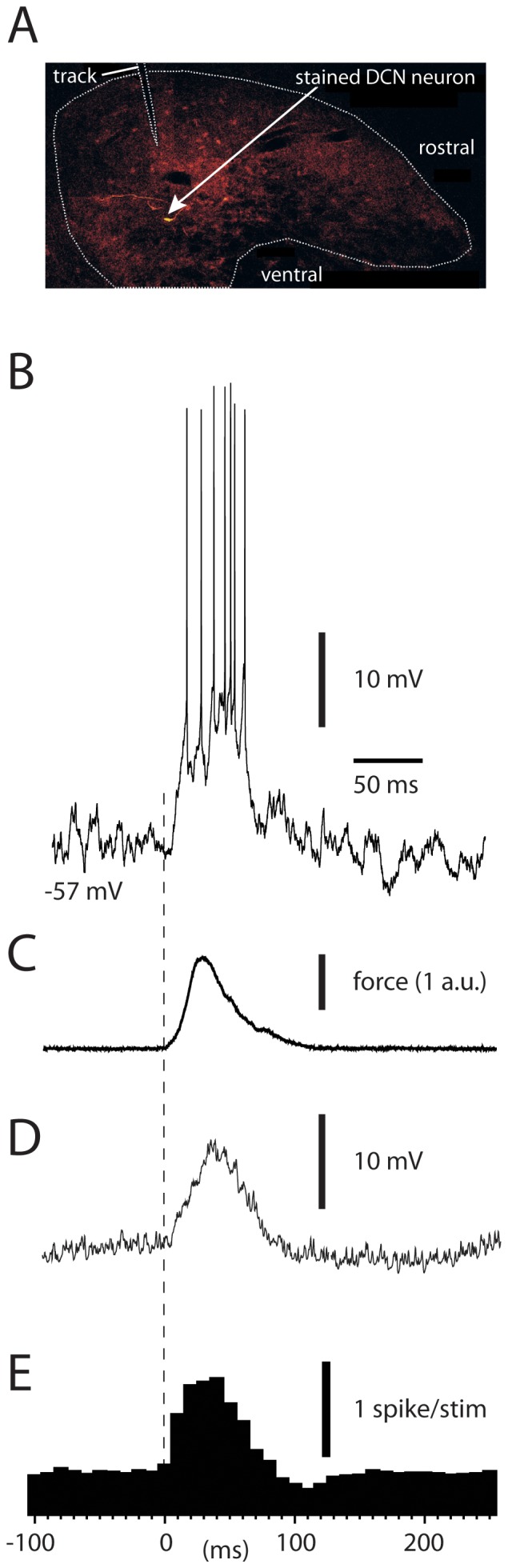
Responses of DCN cells to manual skin stimulation. (A) Location of a recorded, stained DCN neuron in the anterior interposed nucleus. The outlines of the nucleus and the electrode track are indicated with white dotted lines. (B) Sample IC recording illustrating an excitatory response. The neuron was slightly hyperpolarized from resting membrane potential. (C) Strain gauge signal indicating the time course of the force applied during the skin stimulation. (D) Averaged intracellular recording after removing spikes in software from raw data (N = 50 stimulations). (E) Spike responses for the same cell as in (B)–(D) but recorded without hyperpolarization. The bin width of the peristimulus histogram (obtained using N = 50 stimulations) is 10 ms.

Manual skin stimulation could evoke substantial excitatory responses in the DCN cells (Fig. 1A), both in terms of membrane depolarization and in terms of spike responses (Fig. 1B). However, the powerful membrane depolarization evoked by the stimulation was evident (Fig. 1B). In order to obtain comparable responses, manual skin stimulation was monitored as force against time using a strain gauge device mounted on the finger tip of the investigator (Fig. 1C). For quantification, we used responses evoked by stimulations lasting 70–90 ms with comparable peak force magnitudes (1.8+/−0.74 a.u.). Intracellular DCN cell recordings showed that such manual skin stimulation evoked substantial depolarizations with a similar time course as the applied force (Fig. 1B,C). Averaged intracellular responses, obtained from the raw intracellular traces after template-based removal of spikes using software, revealed a straight-forward relationship between the intracellular depolarization and the time-course of the applied force (Fig. 1D). Importantly, in all cells recorded such responses could be evoked without any preceding inhibition (no deviation beyond −1.0 s.d. compared to prestimulus baseline) (N = 8 intracellularly recorded neurons), implying that the excitatory responses were not due to post-inhibitory rebound depolarization. As previously described, due to an extremely high background of PC inhibitory synaptic inputs, and possibly spontaneous MF activity, the baseline membrane potential of DCN cells *in vivo* is extremely noisy [1], which prevented the identification of individual synaptic events. The spike responses of the DCN cells displayed a similar profile as the evoked intracellular responses (Fig. 1E). Overall, the excitatory input consisted of a fast-rising, monotonic net depolarization of the membrane potential with a peak amplitude of 8.8+/−3.3 mV and a net increase in spike output of 97%+/−41% (N = 8). The start of this depolarization (defined as a deviation from prestimulus baseline noise by +2 standard deviations) had an onset of 9.7+/−5.5 ms (N = 8) relative to the onset of the strain gauge signal. Since the strain gauge did not detect contact made with skin hairs, which are known to activate LRN cells [23] and which were inevitably activated before the strain gauge made contact with the skin, this is likely to be a slight underestimation of the response latency time.

**Figure 2:**
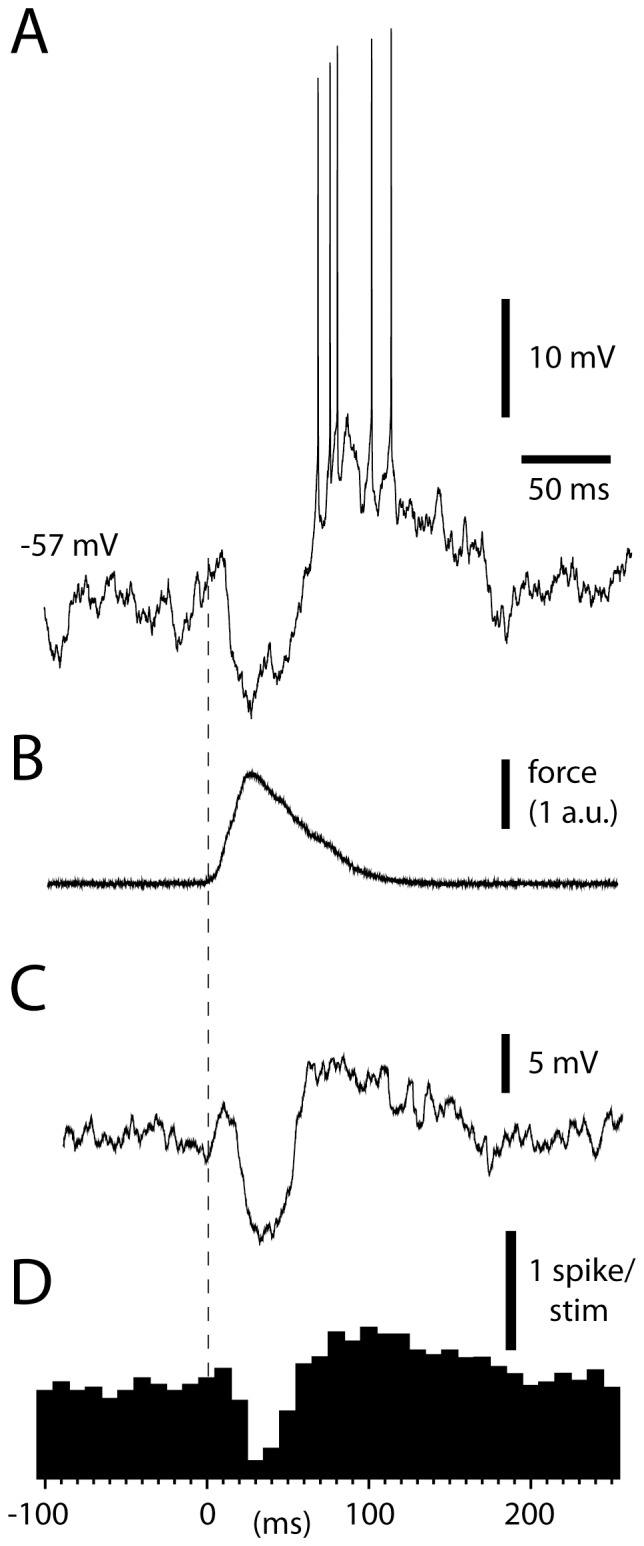
For comparison, responses evoked by a similar stimulation as in Fig. 1 but applied within the CF receptive field of the afferent PCs to the DCN cell (PC-CFRF, see text). Similar display as in Fig. 1.

The fact that these excitatory responses occurred without any preceding inhibition suggested that they were generated by direct MF excitation rather than rebound responses. What would a rebound response look like under these conditions of stimulation? These responses were the focus of a previous paper on the AIP neurons, in which we reported that rebound responses in DCN cells *in vivo* using peripheral stimulation could only be obtained from within the CF receptive field of the afferent PCs (PC-CFRF) [1]. Here, we illustrate an example of a presumed rebound response evoked from the CF receptive field (Fig. 2), primarily for the purpose of contrasting it with the response illustrated in Fig. 1. Notably, there was one fundamental difference from the responses evoked from outside the PC-CFRF: after an initial excitation, the response was cut short by a powerful inhibition, corresponding to the concerted or synchronous CF activation of the afferent Purkinje cells [1], which was in turn followed by an apparent rebound excitation that outlasted the duration of the skin stimulation (Fig. 2).

### Relationship between Excitatory Inputs and the Location of the CF Receptive Fields

**Figure 3:**
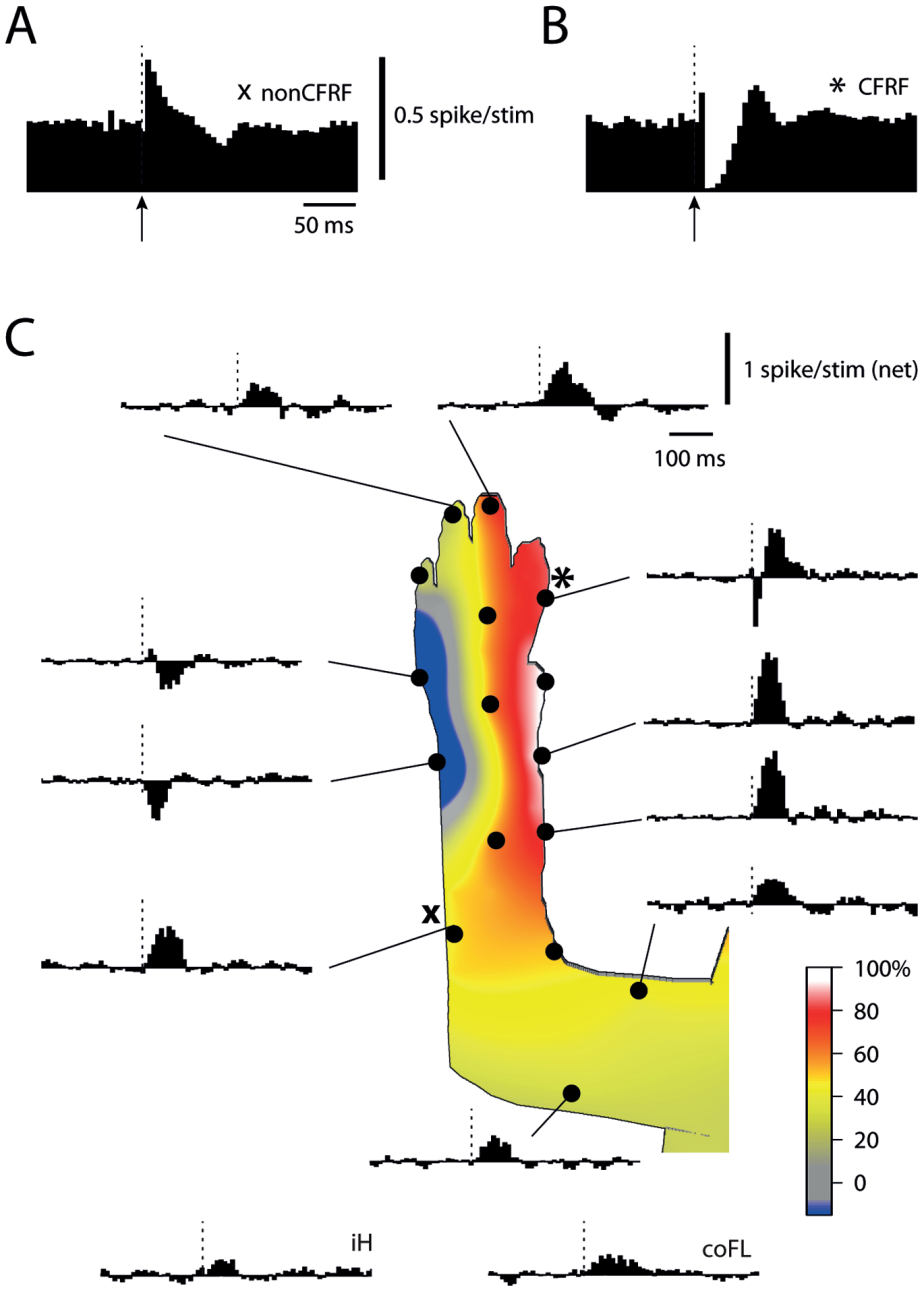
Responses evoked from various skin sites. (A) Spike response evoked by electrical skin stimulation of the skin site indicated by an ‘X’ in (C). Bin width of the peristimulus histograms in A and B, 5 ms. (B) Spike response evoked by electrical skin stimulation within PC-CFRF (asterisk marks the location in C). (C) Histograms of net spike responses evoked from various skin sites. Bin width, 10 ms. iH, ipsilateral hindlimb, coFL, contralateral forelimb (both with a distal location).

We compared the input to the DCN cells evoked from various sites on the skin using manual stimulation. In order to systematize our data, we first identified the location of the PC-CFRF of each DCN neuron using tapping, noxious pinch and electrical skin stimulation as described in a number of previous papers [10], [17], [18]. Figure 3A, B illustrates the differences in responses evoked from outside the PC-CFRF (Fig. 3A) and from within the PC-CFRF (Fig. 3B) using electrical skin stimulation. Using a standard manual stimulation (lasting 70–90 ms, controlled by the strain gauge probe), we repeatedly stimulated a large set of skin sites to obtain a net response histogram for each site (Fig. 3C). Excitatory responses were evoked from all parts of the body, which can be explained by that one of the major afferent pathways to the LRN, the bVFRT system, integrates information from different limbs [31]. However, the input tended to be stronger around and within the PC-CFRF (Fig. 3C). Within the PC-CFRF (site marked with asterisk in Fig. 3C), relatively powerful inhibition was evoked, as would be expected (Fig. 2 and [1]). This inhibition was followed by strong excitation, which in part could have been due to rebound excitation or, alternatively, could be a reflection of a strong underlying MF excitation. Apart from the PC-CFRF, in many DCN cells we could in addition identify a second skin area from which inhibition was evoked, apparantly superimposed on an underlying MF excitation (blue skin area in Fig. 3C). This was also expected since the afferent PCs are not only excited from their CF receptive field but also from a parallel fiber receptive field, which have a specific, non-overlapping location relative to the former [12]–[14]. Given that we could identify the location of the PC-CFRF of the Purkinje cells converging to the DCN neuron, we could use our previously published systematized data for the relationship between the location of CF receptive field and the location of the parallel fiber receptive for the Purkinje cells of the C3 zone [29] to identify a putative parallel fiber-Purkinje cell-receptive field for each DCN neuron (referred to as the PC-PFRF below). In many DCN neurons, stimulation within the PC-PFRF skin area also evoked a moderate inhibitory response, consistent with an increase in simple spike firing of afferent PCs when this skin area was stimulated (this was also shown in Bengtsson et al. [1]). This inhibition was typically mixed with an underlying excitation (Fig. 3C, responses evoked from ‘blue’ skin area). Such inhibitory responses have previously been shown to be eliminated or reduced when the PCs of the cerebellar cortex is blocked, leaving excitatory responses unaffected [32]. In other cells, however, the underlying excitation was stronger and no clear-cut inhibitory response was evoked from the expected PC-PFRF.

**Figure 4:**
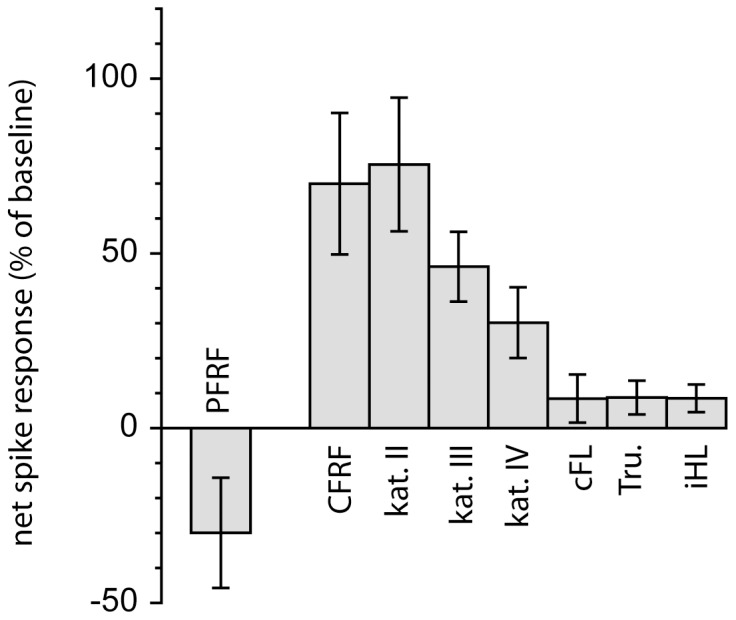
Summary of net spike responses evoked from various skin sites. PFRF, parallel fiber receptive fields of afferent PCs (see text); CFRF, climbing fiber receptive fields of afferent PCs. Kat = category; cFL = contralateral forelimb; Tru. = trunk; iHL = ipsilateral hindlimb.

To systematically compare the DCN responses evoked from different skin locations relative to the location of the receptive fields of the CF and PF inputs from Purkinje cells, we divided the skin sites into 5 categories for all cells recorded (N = 112, Fig. 4). Category I: the CF receptive field (i.e. PC-CFRF); Category II: adjacent to the PC-CFRF, i.e. located on the same limb segment with the same radio-ulnar and/or ventro-dorsal location or on an adjacent digit; Category III: nearby but not adjacent to the PC-CFRF, i.e. on an adjacent segment or on the same segment but with an opposite radio-ulnar and/or ventro-dorsal location; Category IV: Skin sites located on the forelimb but on a non-adjacent segment relative to the PC-CFRF; Category V: verified or putative (based on [14]) PC-PFRF. In this categorization scheme, a segment on the forelimb corresponded to the digits, the paw, the forearm or the upper arm. The borders of segments have previously been shown to be respected in the distribution of CF receptive fields and MF receptive fields of the C3 zone-AIP system [24], [33]. We also measured input from the ipsilateral hindlimb (iH), the contralateral forelimb (coFL) and the trunk (Tru). Figure 4 summarizes the quantified input from these different categories of skin sites. The strongest excitatory DCN responses were evoked from within the PC-CFRF, given that the net excitatory value was dragged down by initial inhibition evoked by CF excitation of the PCs. However, also skin sites located adjacently to the PC-CFRF evoked strong excitatory responses, suggesting that MF input to DCN cells may be particularly strong for skin areas around and perhaps within the PC-CFRF. The level of DCN excitation then gradually declined the further away the skin site was located from the PC-CFRF. One exception, though, was the responses evoked from the PC-PFRF, in which increased Purkinje cell inhibition played a main role in shaping DCN cell responses and which resulted in, on average, a net inhibitory response from the skin areas belonging to this category. Statistical tests (repeated measure ANOVA, followed by *post-hoc* Tukey tests) of the differences in response magnitudes between the groups were significant (p<0.05) for category I versus categories III, IV, V and non-forelimb, respectively, for category II versus categories III, IV, V and non-forelimb, respectively, for category III versus category V and non-forelimb sites, and for category IV versus category V and non-forelimb sites.

### Comparing the Responses of DCN and LRN Cells

**Figure 5:**
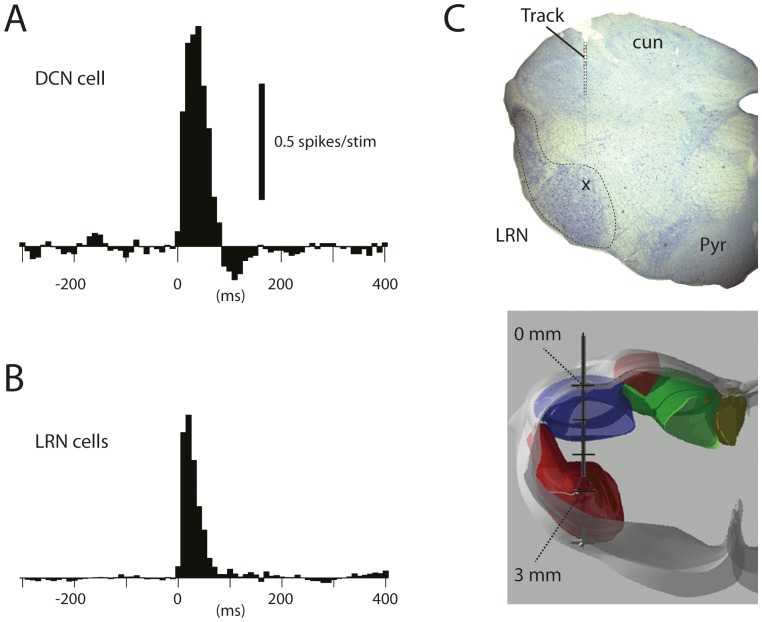
Net spike responses in DCN cells and LRN cells evoked by manual skin stimulation. (A) Net spike response of the same cell as illustrated in Fig. 1. (B) Net spike response shown as an average peristimulus histogram of all LRN cells recorded (N = 29). The motivation for pooling the LRN cells in (B) is that DCN cells would be expected to receive multiple LRN MF synaptic inputs. Bin width in both (A) and (B) is 10 ms. (C) Reconstructed electrode track for a stimulation electrode placed in LRN, shown in a histological section and a 3D reconstruction of the brainstem, respectively.

The LRN is an important source of MF inputs to the AIP, even though input from the direct rostral spinocerebellar tract (RSCT) [19], [20] is also likely to exist. Therefore, we next used manual skin stimulation and recorded the responses of cells in the LRN, to compare them with the excitatory responses of DCN cells. Figure 5 illustrates the net spike responses of a DCN cell (Fig. 5A) (shown for comparison) and the average net response of all LRN cells (Fig. 5B, N = 29). The average net increase in spike output in DCN cells was 4.4+/−1.7 (N = 112, including both intracellularly and extracellularly recorded spike data) spikes per stimulation, which was comparable to that of LRN cells (3.6+/−0.8 spikes per stim, N = 29). The relative net increase in the DCN cell spike responses (+85% +/−34%, N = 112) was an order of magnitude lower than that of LRN cells (+183% +/−37%, N = 29), a difference that at least in part could be explained by the much higher spontaneous firing frequency in DCN cells (34+/−12 Hz, N = 112) compared to LRN cells (9+/−11 Hz, N = 29). In both LRN cells and DCN cells, the onset of all excitatory responses evoked from the forelimb (i.e. for DCN cells only responses evoked from outside the PC-CFRF and the putative PC-PFRF, see below, were considered here) were found to start within the first 20 ms after the onset of the stimulation (defined from the peristimulus histograms as the first bin which exceeded the mean activity of the prestimulus baseline by +1.5 standard deviations). In LRN cells, the average time-to-peak of the responses was 27+/−4.5 ms (N = 29), whereas the responses of the DCN cells peaked at, on average, 42+/−7.2 ms (N = 112). The overall duration of the responses was 72+/−12 ms in LRN cells and 95+/−17 ms in DCN cells. Hence, overall, the time-courses of the responses in the two populations of neurons were comparable, although somewhat faster responses were recorded in the LRN cells.

**Figure 6:**
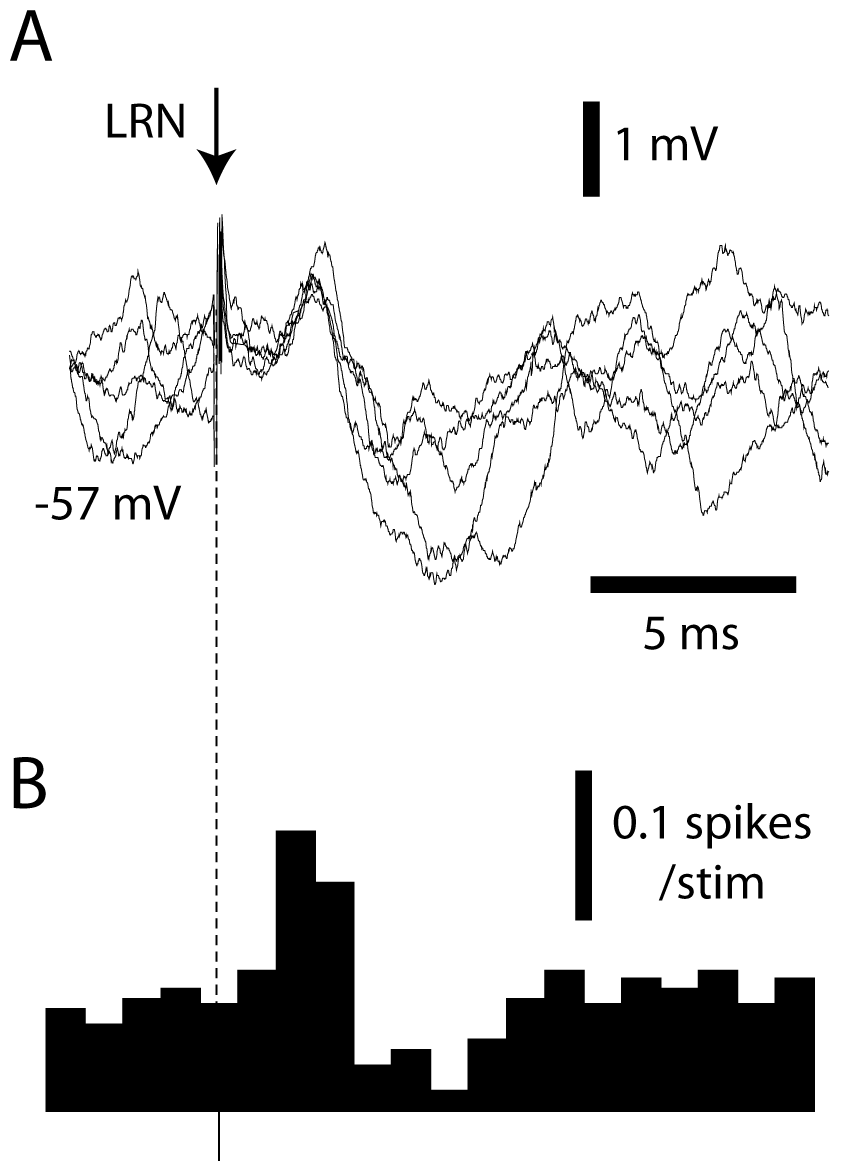
Responses evoked in DCN cells by microstimulaton in LRN. (A) Superimposed raw EPSPs recorded from one DCN cell during hyperpolarization to prevent spiking. LRN stimulation, 50 uA. (B) Histogram of spike responses evoked in the same cell but without hyperpolarization. N = 50 stimulations. Bin width 1 ms.

Manual skin stimulation hence evokes a barrage of spikes in a number of LRN cells. But what would responses evoked by single spikes in the LRN MFs look like in DCN cells? Using a stimulation microelectrode placed in the LRN, EPSPs were evoked in DCN cells at intensities of 10–100 uA. In order to minimize contamination from spike responses, the membrane potential of the recorded DCN cell was lowered using hyperpolarizing currents and the stimulus intensity in the LRN was adjusted to evoke an EPSP response that did not evoke spike responses (Fig. 6A). LRN-evoked EPSPs had a peak amplitude of 0.73+/−0.28 mV and a 10–90% rise time of 0.96+/−0.54 ms (N = 6). Although the synaptic response was relatively modest in amplitude, when the hyperpolarization was removed so that the DCN cell was recorded at resting membrane potential the LRN stimulation consistently evoked spike responses with a net increase in spike output of 0.27+/−0.17 spikes per stimulation (N = 29, including both intracellular and extracellular recordings) calculated from peristimulus histograms (Fig. 6B). Notably, our LRN stimulation never evoked antidromic activation of DCN cells (N = 29), contradicting the conclusions of [34] regarding the existence of a DCN-LRN projection. The relatively large spike increase that we observed, despite the small peak amplitude of the evoked EPSPs, could be explained by a potentially non-linear depolarization-to-spike coupling due to the NMDA receptor-dependent component of the MF responses of DCN cells [35].

## Discussion

The present study provides the first intracellular analysis of natural patterns of excitatory synaptic inputs to DCN cells as well as the first analysis of the spatial relationship between the excitatory cutaneous input to the DCN cells and the cutaneous CF receptive fields mediated by the afferent Purkinje cells. The comparison between the receptive fields of the two afferent sources indicated that the output of the DCN cell targets the same functional components of the motor control circuitry as it samples information from. However, the DCN cells seem to sample information preferentially from more limited motor control circuitry than they control themselves, compatible with a role for the cerebellum in the coordination of large muscle groups.

### The Nature of the Recorded Excitatory Responses

The excitatory responses evoked by manual skin stimulation consisted of a substantial intracellular depolarization, which started essentially immediately after the skin was touched and which was not preceded by inhibition (Fig. 1). Therefore, it is unlikely to be due to the rebound-like intrinsic responsiveness of the DCN cells [36]. In fact, the fast, early excitatory modulations observed are unlikely to be dependent on any activity from the PCs of the cortex, which has also been directly shown by cortical cooling [32]. CF-EPSPs are very weak and completely overruled by the concomitant PC IPSPs [1], [37], leaving the direct MF input to the DCN cells as the only alternative. Accordingly, the neurons of the LRN, one of the main sources of MF inputs to these DCN cells, responded with strong spike responses with an average time course resembling that of DCN cells to the same type of stimulation (Fig. 5). Electrical stimulation of the LRN also evoked EPSPs in DCN cells (Fig. 6) [38], although this more artificial stimulation also evokes a strong inhibition, most likely mediated via the PCs (Fig. 6). Strong excitatory responses in DCN cells that are elicited without any sign of preceding inhibition are also a common finding in behavioral studies [5]–[7]. In addition, retained DCN cell responses after chemical inactivation of GABA receptors, which blocks the cerebellar cortical influence on the DCN cells, support the view that MF inputs have a major role in defining DCN cell output [39].

The manual skin stimulation that we used represents a natural pattern of synaptic input in the sense that it corresponds to a spatiotemporal pattern of skin primary afferent activation and associated activation of multiple parallel afferent pathways [23] that could occur as an external object brushes across that particular skin area. Since the same type of stimulation can strongly activate PCs in the C3 zone [11] the depolarization/excitation that we recorded from many skin areas in each DCN neuron likely reflected inputs that were free of substantial PC inhibition. Inhibition was instead evoked from specific skin areas, corresponding to the PC-CFRFs and the PC-PFRFs, in line with the known organization of this corticonuclear system (see Introduction).

### Inputs to the SCT/SRCT Systems and to CFs from Spinal Motor Circuits

**Figure 7:**
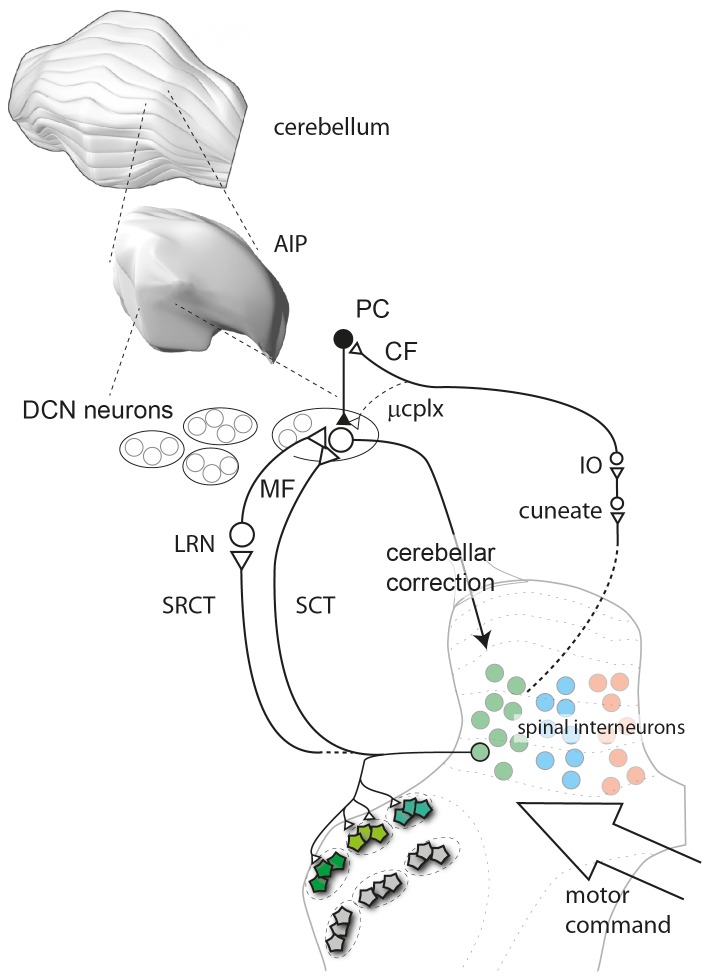
The information conveyed to DCN neurons. The AIP is composed of multiple functional cell groups, each of which forms the output of a cerebellar corticonuclear microcomplex (ucplx). The main mossy fiber input to the DCN cells is derived from spinal motor circuits, which are conveyed to the cerebellum either directly in a spinocerebellar tract (SCT), or via a synaptic relay in the LRN, in a spino-reticulo cerebellar tract (SRCT). These tracts are in turn driven by spinal interneurons, which target specific combinations of muscles or synergies. Pentagons in the ventral horn are alpha-motorneurons encircled by dashed lines to indicate separate spinal motor nuclei, and each spinal interneuron targets a limited number of motor nuclei [58], [59] as indicated by example. Hence, the information carried in SCTs/SRCTs informs the DCN cells of the final composition of the motor command, i.e. which muscle synergies that are currently driven. Also the input that drives the CFs is derived from the spinal motor circuitry (indicated by dashed line from the spinal interneurons), via relays at least in the cuneate nucleus and the inferior olive (IO). The spinal motor circuitry is driven by motor commands from the neocortex and the brainstem. The output of the DCN cell represents the cerebellar correction of ongoing motor commands and targets the neocortex and the brainstem. Since the CF receptive field identifies the microcomplex the DCN cell is located in and consequently the muscle synergy it is connected with, comparing the SCT/SRCT input with the location of the CF receptive field is equal to comparing the motor information it receives with the motor information it issues. Accordingly, our findings suggest that the input and the output of the DCN cell are functionally matched. AIP, Anterior interposed nucleus; PC, Purkinje cell; CF, climbing fiber; ucplx, microcomplex; MF, mossy fiber; IO, inferior olive; LRN, lateral reticular nucleus; SRCT, spinoreticulocerebellar tract; SCT, spinocerebellar tract.

The cells of the LRN, and possibly also the RSCT, which provides MFs to the anterior interposed nucleus [40], [41], are driven by synaptic inputs from spinal neurons [22], [42], which integrate sensory and motor information (see Spanne and Jorntell [43]) (Fig. 7). For example, the population of C3–C4 propriospinal neurons, which plays a critical role in forelimb motor control, is an important source of input to the LRN [42], [44], [45]. However, LRN also receives input from spinal neurons located below the spinal segment C5 [22], [46], and the RSCT possibly mainly originates from neurons located below C5 [20]. It is presumably these neurons that mediated the potent excitation from distal forelimb skin [23] since the C3–C4 propriospinal neurons do not receive such input [47]. Also the input to the rostral dorsal accessory olive, which supply CFs to the forelimb region of the C3 zone [16] are mediated via spinal neurons, specifically the neurons of the postsynaptic dorsal column (PSDC) pathway [48], [49], which ascend in the dorsal funiculus, make synapses in the cuneate nucleus, before the information reaches the inferior olive (Fig. 7).

### A Note on the Choice of Preparation

The decerebration of our preparation naturally led to a loss of afferent pathways involving the cerebral cortex, most notably the cortico-pontine input. However, since the spino-cerebellar systems, the LRN and the transmission through the cerebellar cortex are severely depressed by anesthetics, decerebration was a necessary step to maintain as normal processing as possible within these systems while still allowing the mechanical stability required for the deep patch clamp recordings. However, despite the loss of the neocortical inputs we note that the range of both DCN cell activation and PC activation is well within the range of maximal modulations reported from animals under behavior [6] [5] [50], [51]. The fact that we worked with a decerebrate preparation can explain why our responses have a straight-forward, monophasic character: Rowland and Jaeger [52] concluded that late components in responses evoked by tactile stimulation was due to activation of circuitry loops across the neocortex.

### Functional Implications of the Relationship between Excitatory Inputs and CFs in DCN Cells

We found that the cutaneously evoked excitatory inputs to the DCN cells had a gradient of increased sensitivity towards the PC-CFRF. Importantly, the PC-CFRF of a DCN neuron reflects its function since multi-segmented movements can be evoked by microstimulation of an AIP cell group and this movement withdraws the CF receptive field of the cell group [17]. In addition, cutaneously driven spinal interneurons of the lumbar spinal cord in the rat have been shown to have a similar input-output relationship as the DCN neuron, i.e. withdrawal of the receptive field, although the movements controlled by spinal interneurons are confined to fewer segments of the limb [25], [26] and possibly fewer muscles at each segment. Since we here found that the most powerful excitatory input came from skin areas located nearby the PC-CFRF of the DCN neuron, the input and output of the DCN neuron appears to be functionally matched. In other words, the DCN neuron is preferably excited by spinal interneuron circuitry which has a similar motor function as the DCN neuron (Fig. 7). This finding is in agreement with the anatomically defined connections in the closed cerebello-neocortical loops [53], [54], although our findings relate to functionally defined circuitry rather than anatomically defined. A difference from the anatomical findings, however, is that the DCN neuron seems to control a wider set of synergies [17], [18] than the single spinal interneuron [55]. In addition, the individual DCN neuron also received weaker input from wide regions of the skin, suggesting that it samples input from very large numbers of spinal interneurons controlling diverse sets/synergies of muscles. This would allow the DCN neuron to sample information from a weighted average of a number of spinal synergy controllers and to use that information to regulate the activity in the more complex synergies it controls.

### Potential Mechanisms for Establishing the Specificity

As each DCN neuron could receive up to 100’s of MFs [1] with small receptive fields [23], the relationship we found with a gradient of increased excitatory input from skin sites the closer they were located to the PC-CFRF probably reflects that the MF synapses have different synaptic weights depending on the skin area they are activated from. How could this differential synaptic weight distribution arise? Two alternatives, which are naturally not mutually exclusive, exist; hardwiring from development or refinement of synaptic weights through learning or plasticity. If the information the MFs and the CFs convey has its origin in the spinal circuitry as discussed above, proximity in receptive field locations would mean a close functional relationship as the receptive fields of spinal neurons reflect their function in terms of muscle activation [56]. Importantly, spinal circuitry is not statically generated by genetic codes but subject to substantial modifications well after birth if the actions of the muscles it innervates change [57]. Hence, the spinal circuitry is adapted to the precise biomechanics and anatomy of the musculoskeletal apparatus, which may change over time. Viewed against this background, it seems likely that the specificity between the SCT/SRCT-RF and the PC-CFRF in the DCN neurons are established, and possibly fine-tuned over time, at least partly through plasticity at the MF-to-DCN synapses. Future studies are needed to further explore whether the mf-DCN relationship could change through learning also in adult life – the consequences for movement coordination and the ability to learn new complex movements could be pivotal.
